# Combination liposomal amphotericin B, posaconazole and oral amphotericin B for treatment of gastrointestinal *Mucorales* in an immunocompromised patient

**DOI:** 10.1016/j.mmcr.2017.05.004

**Published:** 2017-05-24

**Authors:** Anthony Anderson, Dayna McManus, Sarah Perreault, Ying-Chun Lo, Stuart Seropian, Jeffrey E. Topal

**Affiliations:** a Department of Pharmacy, Yale New Haven Hospital, 20 York Street, New Haven, CT 06510, USA; b Department of Pathology, Yale School of Medicine, 20 York Street, New Haven, CT 06510, USA; c Department of Internal Medicine, Hematology Section, Yale New Haven Hospital, 20 York Street, New Haven, CT 06510, USA; d Department of Internal Medicine, Infectious Disease Section, Yale New Haven Hospital, 20 York Street, New Haven, CT 06510, USA

**Keywords:** Oral amphotericin B, Gastrointestinal *Mucor*

## Abstract

Mucormycosis is a life threatening infection caused by fungi in the order Mucorales. Mucormycosis can affect any organ system with rhino-orbital-cerebral and pulmonary infections being the most predominant infection types. Gastrointestinal mucormycosis is rare and accounts for only 4–7% of all cases. Here, we present a case of invasive gastrointestinal mucormycosis in an immunocompromised host treated with systemic and topical anti-mold therapy.

## 1 Introduction

Mucormycosis is a life threatening infection caused by fungi in the order Mucorales. *Rhizopus*, *Mucor*, and *Rhizomucor* species account for up to 75% of mucormycosis cases in patients with hematologic malignancies [1]. Traditional risk factors for mucormycosis are similar to other opportunistic mold infections and include prolonged neutropenia, high dose corticosteroids, severe Graft Versus Host Disease (GVHD), high risk stem cell transplantation (matched unrelated, haploidentical donor, cord blood and T-cell depleted stem cell transplant) and use of *Aspergillus* directed prophylactic agents such as voriconazole or echinocandins [1]. Mucormycosis can affect any organ system with rhino-orbital-cerebral and pulmonary infections being the most predominant infection types [2], [3]. Gastrointestinal mucormycosis is rare and accounts for only 4–7% of all cases [2]. The stomach is the most common site of infection, followed by colon, small intestine and esophagus [4]. Treatment traditionally consists of surgery and liposomal intravenous amphotericin B. Posaconazole, an azole with activity against some mucorales species has also been utilized often in combination with amphotericin B and surgery [4], [5]. Despite aggressive treatment regimens, mortality rates remain high in stem cell transplant patients with reported rates at 75% [6], [7]. We present a case of invasive gastrointestinal mucormycosis in an immunocompromised host treated with systemic and topical anti-mold therapy.

## 2 Case

A 43-year-old male with a history of tyrosine kinase inhibitor-resistant CML underwent a fully matched unrelated donor peripheral blood hematopoietic stem cell transplant (HSCT) following conditioning with high dose pharmacokinetic guided busulfan plus fludarabine, and anti-thymoglobulin. The patient presented on Day 0 (Day 68 from transplant) with abdominal pain, fever and severe diarrhea. A computed tomography (CT) scan of the abdomen and pelvis demonstrated diffuse wall thickening and mucosal enhancement involving the distal small bowel, consistent with enteritis. Stool was tested for *C. difficile*, Adenovirus, Enterovirus, Norovirus, Rotavirus and parasites, all with negative results. Esophagogastroduodenoscopy (EGD) and flexible sigmoidoscopy was performed with biopsies of the duodenum and stomach demonstrating severe gastrointestinal (GI) GVHD overall grade 4, stage 4. Treatment of the GI GVHD consisted of methylprednisolone 2 mg/kg/day, infliximab with basiliximab, and budesonide starting on Day 3. The patient was concurrently started on antifungal prophylaxis with voriconazole 200 mg orally twice daily with the initiation of methylprednisolone. On Day 9, a steady state voriconazole trough concentration level was drawn, which was below therapeutic goal at 0.6 mcg/mL (goal 1–4 mcg/mL). The voriconazole dose was increased to 250 mg orally twice daily on Day 15, and a repeat steady state voriconazole trough level was drawn on Day 21 revealed a therapeutic level of 1.7 mcg/mL.

**Fig. 1:**
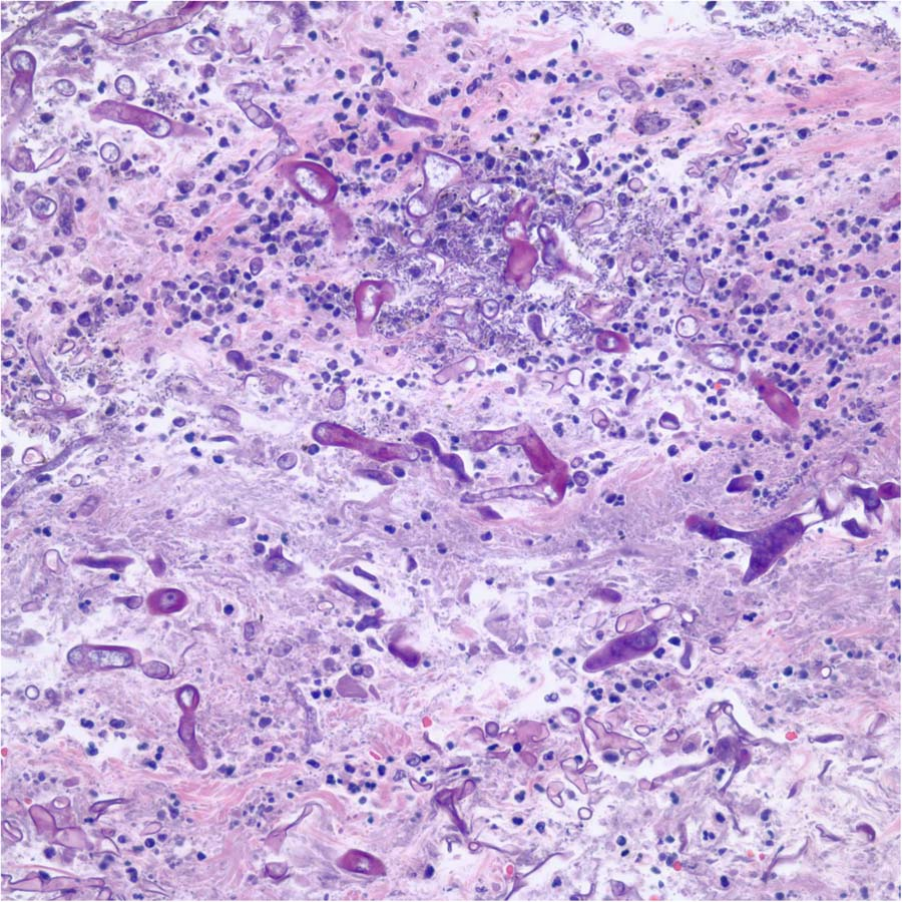
Biopsy of tongue: Numerous broad-based fungal organisms are found in the deep soft tissue of the anterior tongue. The morphology of the fungus is consistent with *Rhizopus* species.

**Fig. 2:**
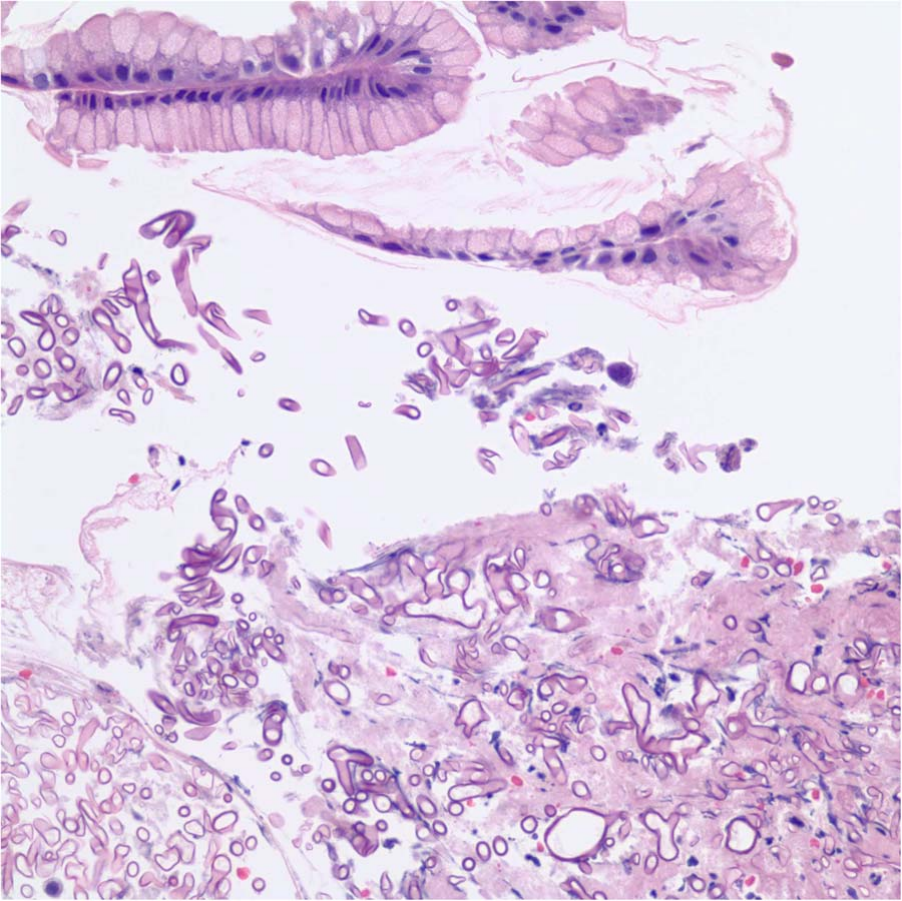
Biopsy of stomach: Biopsy of the antral ulcer in the stomach shows *Mucorales* within gastric mucosa. No definitive evidence of graft versus host disease is seen.

After Day 20 of high dose corticosteroids, the patient developed a black lesion on the anterior portion of the tongue. On Day 22, cultures and a biopsy of the tongue were obtained which revealed *Rhizopus microsporus* by gene sequencing (Fig. 1). Susceptibilities preformed on the culture revealed a posaconazole MIC of 0.5 mcg/mL and isavuconazole MIC of 2 mcg/mL. A CT scan of the head and sinus showed no acute abnormalities. The lesion was debrided and antifungal treatment was switched from voriconazole to intravenous liposomal amphotericin B 3 mg/kg based on the patient's actual weight on Day 25. Monthly intravenous immunoglobulin replacement for hypogammaglobulinemia was concurrently given with treatment. Due to continued diarrhea, a repeat EGD and flexible sigmoidoscopy was performed on Day 29 showing improvement of GVHD, but *Mucorales* was detected within the mucosa of a gastric antral ulcer by histopathology (Fig. 2). Tissue biopsies obtained from repeat EGDs on Day 44 and 59 demonstrated persistent *Mucorales* infection associated with multiple gastric ulcers. No evidence of *Mucorales* was found in the ileum or the colon. The fungal culture of the gastric lesions did not reveal any viable organisms which definitive speciation could not be performed. However, it was assumed they were of the same species due to the GI tract involvement.

Posaconazole delayed released tablets of 300 mg orally every 12 hours × 2 doses, then 300 mg orally daily was empirically added to the intravenous liposomal amphotericin B therapy for refractory mucormycosis on Day 58. Posaconazole steady state trough concentration level was drawn on Day 64 with a level of 340 mcg/mL (goal > 700 mcg/mL). The posaconazole dose was increased to 400 mg orally daily on Day 70 and a steady state posaconazole level repeated on Day 79 was 510 mcg/mL. The posaconazole dose was increased a second time to 600 mg orally daily on Day 85 with a therapeutically level of 780 mcg/mL drawn on Day 90. Recurrent gastrointestinal bleeding continued with repeat EGD/colonoscopy not performed due to the location of the acute bleed. A capsule endoscopy was performed instead which showed portal gastropathy, diffusely irregular small intestinal mucosa and small intestinal mass with associated ulcers and bleeding concerning for persistent *Mucorales* infection.

Given the persistent *Mucorales* infection, oral amphotericin B suspension 100 mg/mL concentration was given as 10 mL by mouth three times a day for its non-absorbable properties to apply local therapy to the site of the infection starting on Day 74. The patient became persistently febrile on Day 80 despite broad spectrum antibiotics. New pleural effusions and ground glass opacities were seen on CT scan. Epstein Barr Virus (EBV) was detected in the blood at 3.52 log which increased to 4.42 log by Day 93. Bronchoscopy was performed with no opportunistic infections identified on culture or cytology. He continued to have progressive tachycardia, hypoxemia with bilateral pleural effusions, and febrile episodes with no clear etiology. Procalcitonin and B-D glucan assays were negative. Liver ultrasound was performed due to hyperbilirubinemia which revealed a large number of masses in the liver of unknown etiology. Intravenous amphotericin B, oral amphotericin B and posaconazole was discontinued on Day 93. The patient continued to have recurrent pleural effusions, worsening hypoxia, fevers and liver masses from which he succumbed to on Day 96 (Day 164 from transplant).

**Fig. 3:**
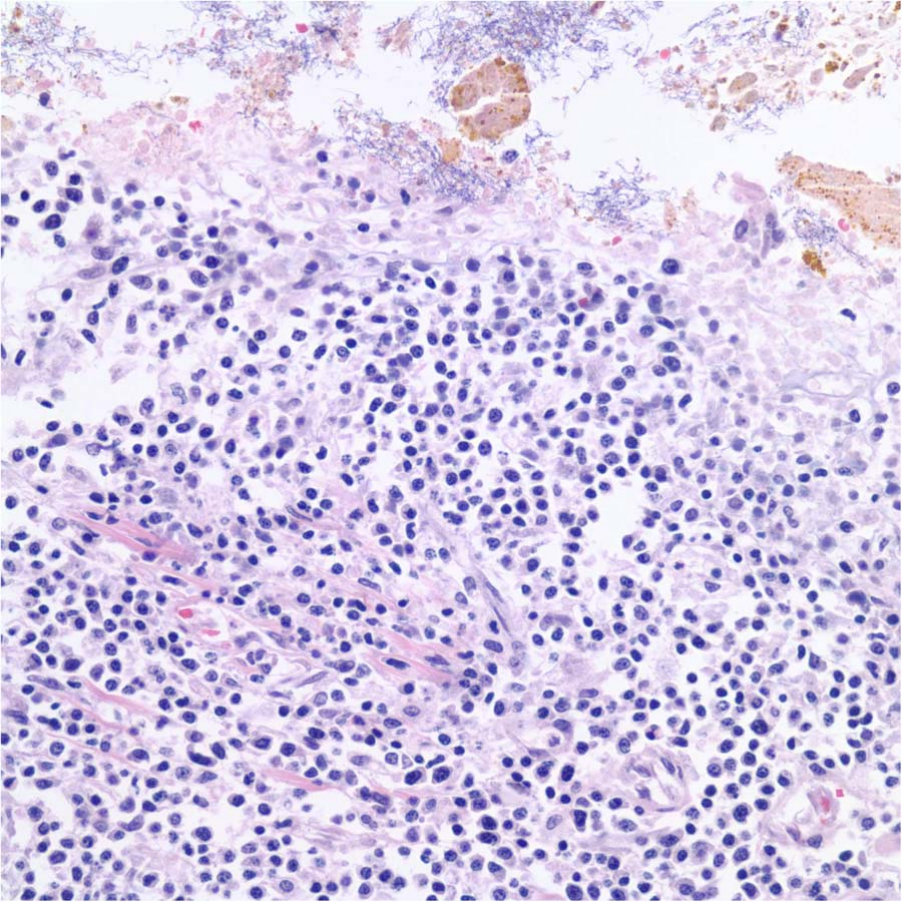
Small bowel histology at autopsy: Histological examination of the gastroenteric ulcers shows aggregation of polymorphic lymphocytes, suggestive of low-grade post-transplant lymphoproliferative disorder. No residual *Mucorales* or graft versus host disease is identified.

**Fig. 4:**
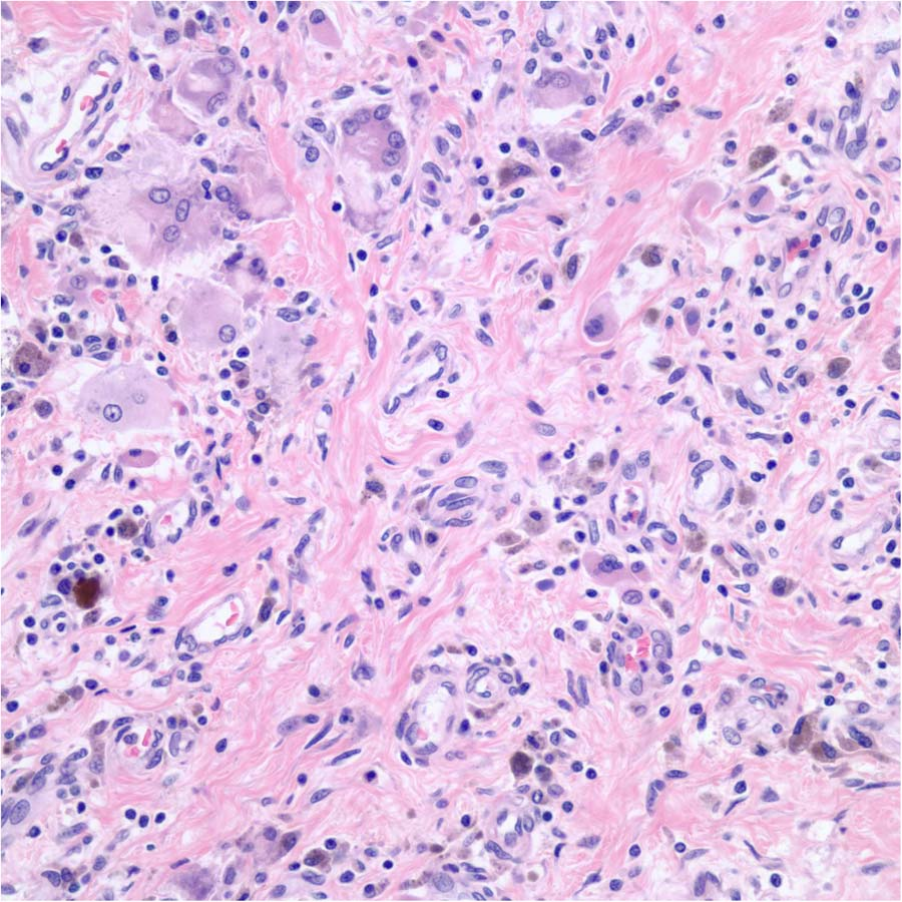
Tongue histology at autopsy: At autopsy, histological examination of the tongue shows multinuclear giant cell reaction, fibrosis and hemosiderin deposition, consistent with repairing changes. No residual *Rhizopus* is seen.

At autopsy, the final diagnosis of the terminal cause of death was acute multifocal interstitial pneumonia with focal organizing pneumonia in combination with EBV related post-transplant lymphoproliferative disorder along the small intestine and liver (Fig. 3). *Mucorales* was not detected from the gastrointestinal tract or on the tongue (Fig. 3, Fig. 4).

## 3 Discussion

Gastrointestinal mucormycosis is a rare, often fatal, invasive infection affecting immunocompromised hosts. A substantial number of gastrointestinal mucormycosis cases are associated with mucositis and neutropenic enterocolitis likely due to breakdown in the intestinal mucosa. Additionally the use of non-*Mucorales* active, antifungal prophylaxis predisposes patients to invasive fungal disease [4]. Mortality rates of up to 75% are reported in HSCT patients despite aggressive therapy [6], [7]. Management of GI mucormycosis consists of reversal of underlying predisposing risk factors if possible, surgical debridement, and effective anti-fungal treatment [1], [4].

Primary anti-fungal therapy for GI mucormycosis is a polyene with liposomal intravenous amphotericin B preferred over conventional amphotericin B because of penetration into the gastrointestinal tract and favorable toxicity profile [2], [4]. High dose liposomal amphotericin B is not recommended due to greater adverse effects with no benefit in efficacy compared to the 3 mg/kg daily dose [8]. Therapy should be initiated within a week of diagnosis; delays in therapy result in poorer outcomes [9]. The role of combination therapy is unclear with no evidence available for GI mucormycosis. Salvage therapy has focused on single agent posaconazole with a retrospective series showing 60–70% partial or complete response in invasive *Mucorales* [5]. It is unclear if these reports translate to GI mucormycosis where posaconazole solution or delayed released tablet efficacy could be attenuated by poor absorption [5]. In our patient, the standard of care approach included liposomal intravenous amphotericin B and debridement. Double coverage with posaconazole was started when repeat biopsies still revealed *Mucorales*. Poor absorption of the posaconazole tablets was seen with our patient requiring two dose adjustments to get to therapeutic goal, indicating the extent of the destruction of the GI tract from the GVHD. Given the *Mucorales* persisted in the GI tract, we considered that a topical antifungal agent could be beneficial, due to unknown penetration or absorption of medications into the GI tract.

Amphotericin B oral suspension previously has been used topically for the treatment of fluconazole refractory esophageal candidiasis and resistant *Candida* species [10], [11], [12]. Amphotericin B oral suspension is < 10% systemically absorbed allowing maximum concentration in the GI tract. Due to the poor systemic absorption, adverse effects routinely seen with the intravenous formulation, such as renal dysfunction and electrolyte abnormalities have not been reported. The most common adverse effect reported is diarrhea, which was closely monitored due to our patient's history of having GI GVHD. The suspension was compounded with amphotericin bulk powder with sterile water to a concentration of 100 mg/mL where the patient received 10 mL (1000 mg) swish and swallow by mouth three times a day. The dosing utilized was based on data for treatment of *Candida* esophagitis (500 mg swish and swallow three to four times a day), fungal prophylaxis in neutropenic patients (1000 mg swish and swallow three times a day along) and *in vitro* susceptibilities of *Zygomycota* to polyenes [10], [11], [12], [13], [14]. Duration of therapy was to be based on repeat EGDs.

Amphotericin B oral solution has the potential to be an effective component of combination therapy for localized GI mucormycosis. Although most of the data of treating GI mucormycosis utilized intravenous amphotericin B with surgical debridement, the addition of oral amphotericin B as adjuvant therapy may be beneficial. Based on our case report, noting clearance of *Rhizopus* from the tongue and *Mucorales* from the GI tract on pathologic exam, we conclude that the use of oral amphotericin could be and effective addition in combination with standard therapy for this difficult to treat infection.

## Conflict of interest

The authors declare that there is no conflict of interest regarding the publication of this paper.
